# Supplementary material for: A STEDable BF2-Azadipyrromethene Fluorophore for Nuclear Membrane and Associated Endoplasmic Reticulum Imaging
Source: Membranes (Basel). 2025 Jan 1;15(1):9. doi: 10.3390/membranes15010009 (PMC11766805; doi:10.3390/membranes15010009)
Supplement: Supplementary file 1 [file membranes-15-00009-s001.zip › membranes-3355069-supplementary.pdf]

A STEDable BF_2-azadipyrromethene fluorophore for nuclear membrane and associated endoplasmic reticulum imaging

Anaïs C. Bourgès [1,*], Massimiliano Garre [1], Dan Wu [1] and Donal F. O'Shea [1,*]

[1]Department of Chemistry, RCSI, 123 St Stephen's Green, Dublin, D02 YN77, Ireland;

*Correspondence: donalfoshea@rcsi.ie; anaisbourges@rcsi.ie

Table of contents:

Movie S1: STED 3D stack of fixed HeLa cells stained with NM-ER. Image size is 45x45x8 µm (xyz).

Movie S2: Eight-hour h timelapse (an image every 7 min) of HeLa cells stained with NM-ER. Overlay of the brightfield and fluorescence.

Movie S3: Confocal 3D stack of multiple live MDA cells co-stained with Hoechst and NM-ER. Image size is 116x116x14 µm (xyz).

Movie S4: Confocal 3D stack of multiple live HeLa cells co-stained with Hoechst and NM-ER. Image size is 76x76x8 µm (xyz).

Figure S1: Co-localization comparison of NM-ER with ER-Tracker Green.

Figure S2: Photobleaching resistance of NM-ER.

Figure S3: 3D STED images of fixed HeLa stained with NM-ER.

Figure S4: Fixed HeLa cells co stained with NM-ER **1** and Hoechst.

Figure S5: Fixed MDA (**a-b**) and HeLa (**c-e**) cells co-stained with WGA-CF594 and Hoechst.

Figure S6: Fixed and permeabilized HeLa cells co-stained with antibodies against Lamin B coupled with CF594 and Hoechst.

Figure S7: Fixed and permeabilized HeLa cells co-stained with NM-ER and LaminB-CF594.

Figure S8: Analytical spectra for **1.**

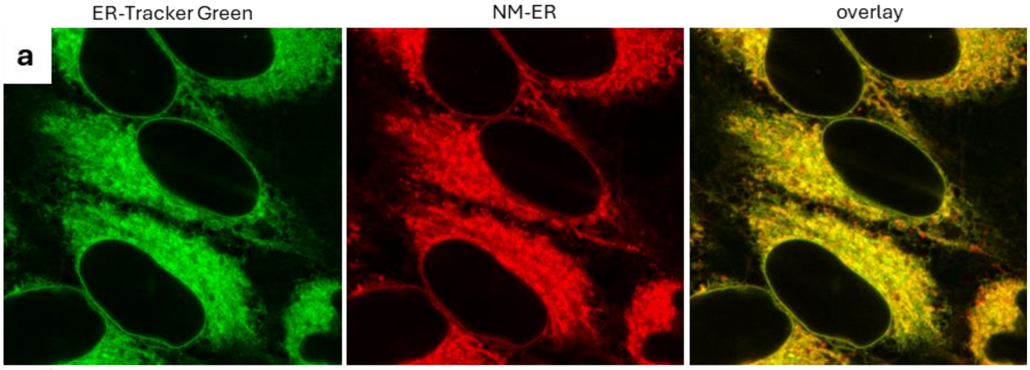

b

Person's coefficient	Mander's coefficient using threshold	
r	M1	M2
0.90 ± 0.02	0.90 ± 0.04	0.89 ± 0.03

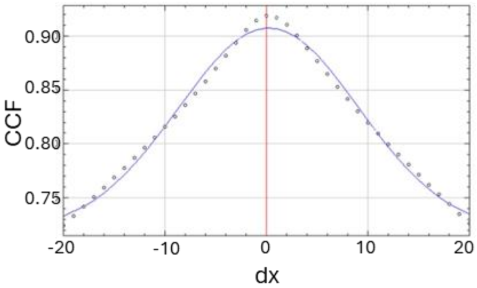

Figure S1: Comparison of NM-ER with ER-Tracker Green. (**a**) Live HeLa cells co-stained with the commercially available ER stain ER-Tracker Green (in green) and NM-ER (in red). The colocalization of the 2 stains (yellow on the right image) were evaluated using 8 different FOVs and the ImageJ plug-in JACoP (**b**). The different co-localization indicators (average of the 8 FOVs in the table and one representative diagram of the overlap coefficient) indicate a very high colocalization of the two.

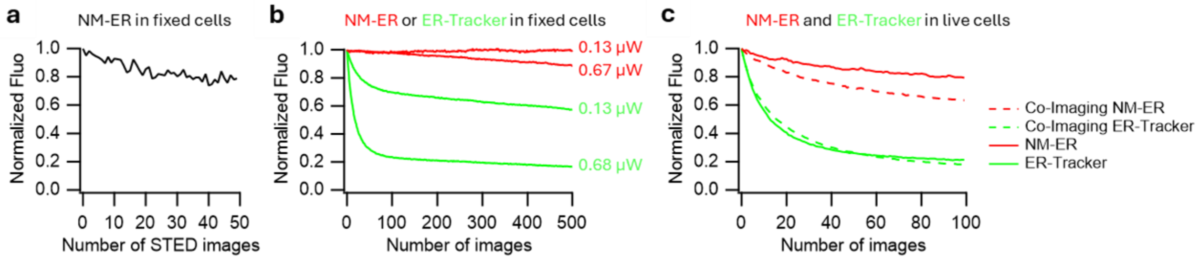

Figure S2: Photobleaching resistance of NM-ER. (**a**) Fixed HeLa cells stained with NM-ER. The same FOV was imaged 50 times using the same STED parameters and the average intensity of the same ROI plotted overtime. The excitation was set at 0.26 µW and the depletion laser at 0.21mW with an exposure time of 5.6 µs / pixel (using line accumulation 3). (**b**) Fixed HeLa cells stained with NM-ER or ER-Tracker Green. The same FOV was imaged 500 times using the same parameters except the excitation wavelength (500 or 594 nm). Two different powers of the WL laser were used corresponding to the minimum (0.13 µW) and the maximum power (0.68 µW) used for cell imaging during this work. (**c**) Live HeLa cells co-stained with NM-ER and ER-Tracker Green. The same FOV was imaged 100 times with both excitation wavelengths (dotted lines) or either one or the other (continues lines) set at 0.67/0.68 µW.

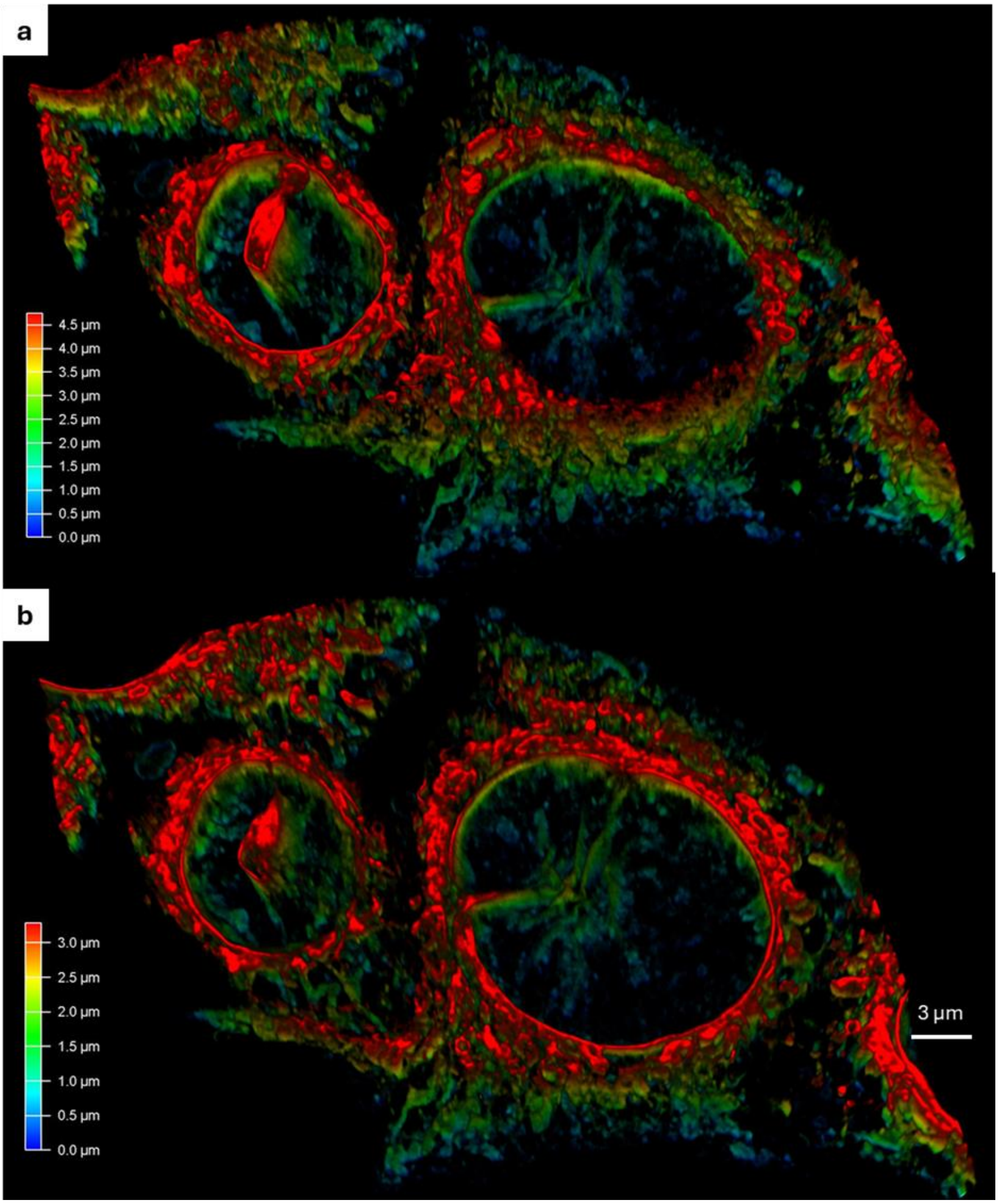

Figure S3: 3D STED images of fixed HeLa stained with NM-ER. (**a-b**) 3D image with the depth color coded with 0 µm corresponding to the bottom of the cell near the coverslip (blue) and the top of the cell is in red. The original movie of the different z planes can be found as Movie S3 and was acquired over 8 µm. In (**a**) images from 4.8 µm to the top (8 µm) were removed to visualize the inside of the nucleus with invaginations and the NM / ER. The same display was done for (**b**) with even more images removed.

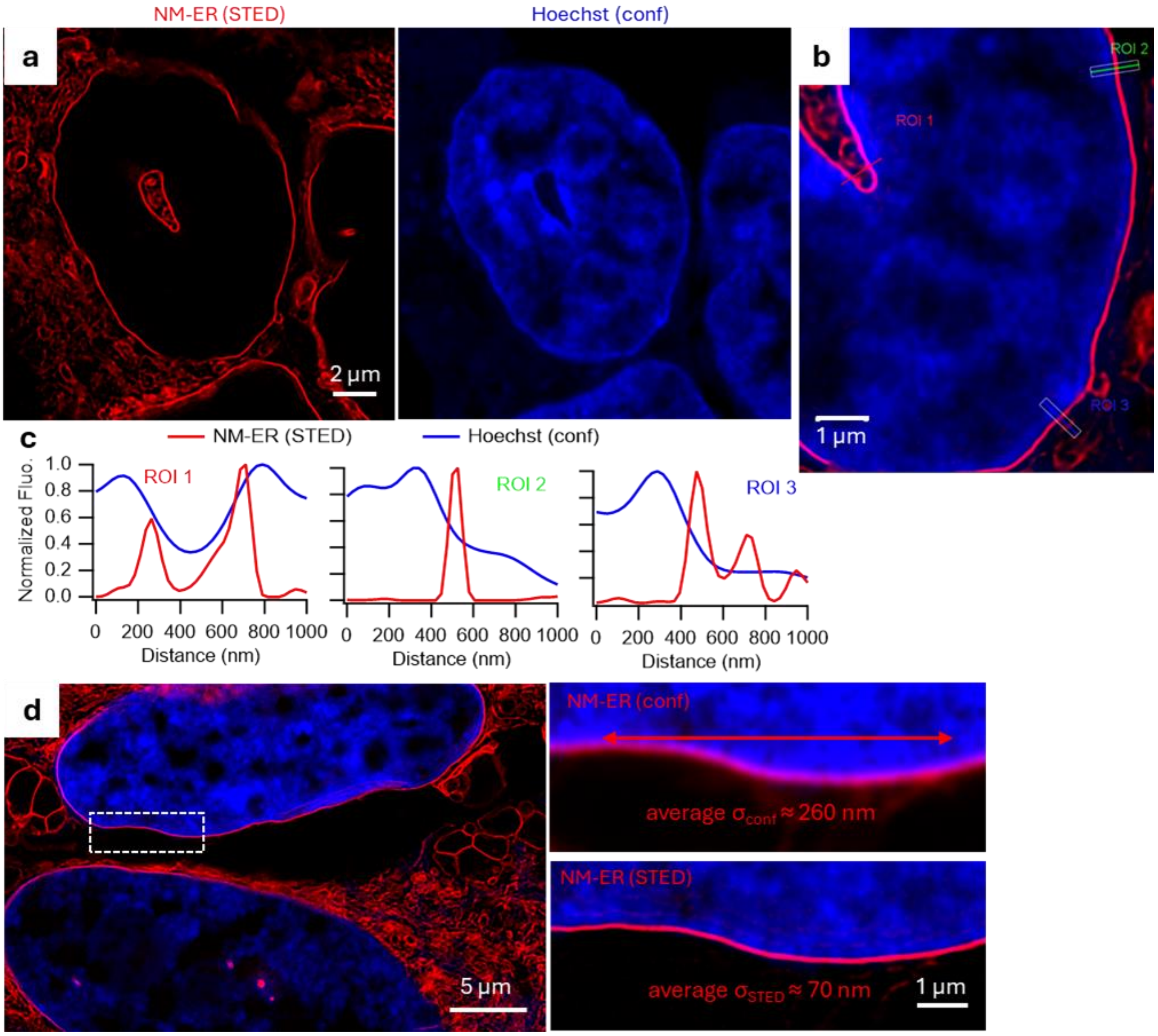

Figure S4: Fixed HeLa cells co-stained with NM-ER **1** and Hoechst. (**a**) The two detection channels NM-ER in STED (red, left) and Hoechst in confocal excitation (blue, right) and (**b**) a zoom in of the overlay with different ROIs. The line profiles of each of them are plotted in (**c**). (**d**) Overlay of a different region (left image) and an enlargement of the nucleus envelope with comparison between confocal excitation (top image) and STED (bottom image). Average σ is the average FWHM measured for multiple selection of the nucleus membrane selected within the region indicated by the red arrow.

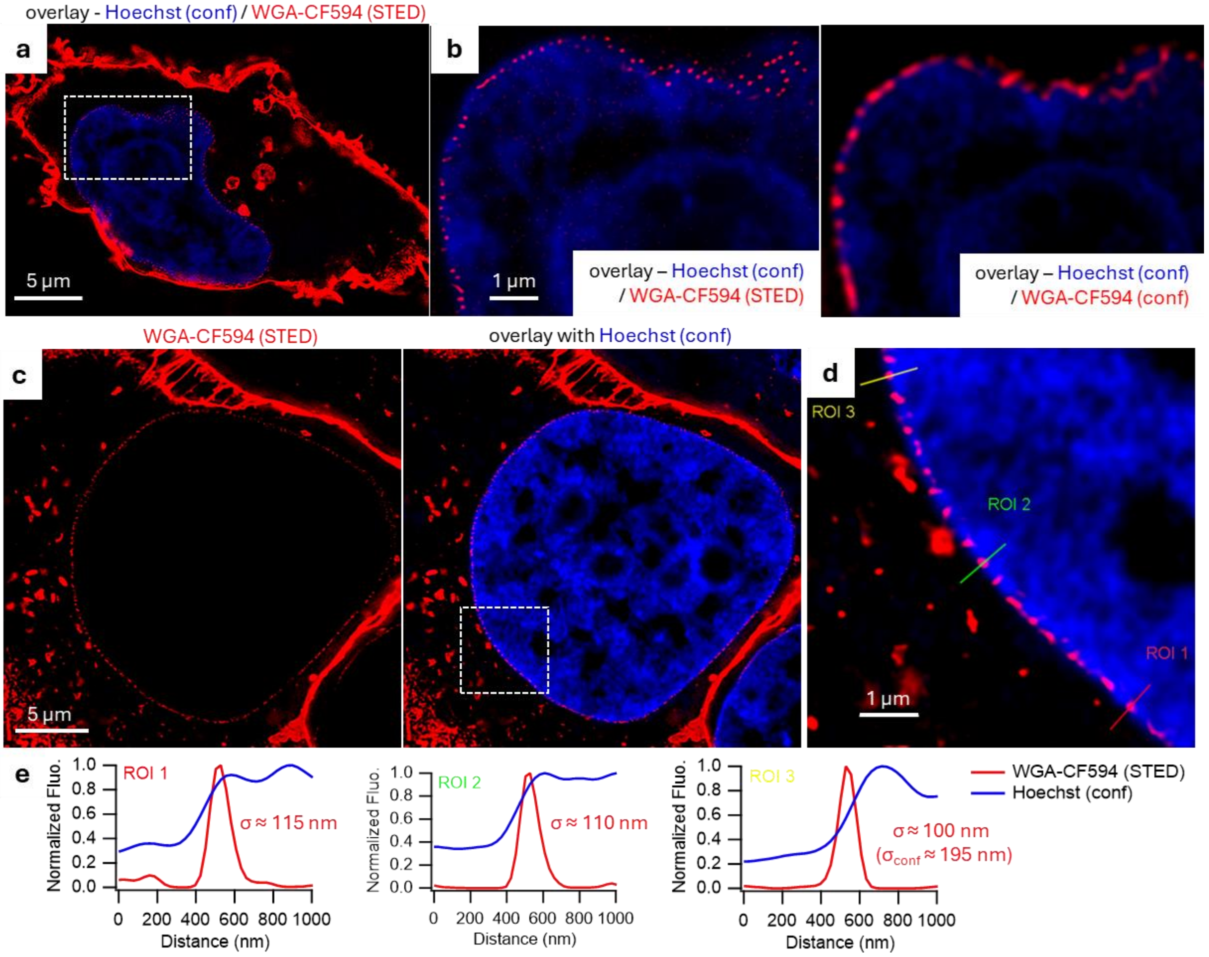

Figure S5: Fixed MDA (**a-b**) and HeLa (**c-e**) cells co-stained with WGA-CF594 and Hoechst. (**a**) Overlay of the two detection channels WGA-CF594 in STED (red) and Hoechst in confocal excitation (blue, conf) and (**b**) an enlargement of the overlay with comparison of WGA-CF594 with STED (left image) and confocal (right image) excitation. (**c**) WGA-CF594 STED image (red, left image) and overlay with Hoechst in confocal (right image). (**d**) Expansion of the overlay with ROIs. The line profiles of each are plotted in (**e**). σ is the FWHM of each ROIs and corresponds to WGA-CF594 "aggregate" located in a single nuclear pore (average measured for 20 pores: σ_{conf} = 196 ± 16 nm and σ_{STED} = 92 ± 15 nm).

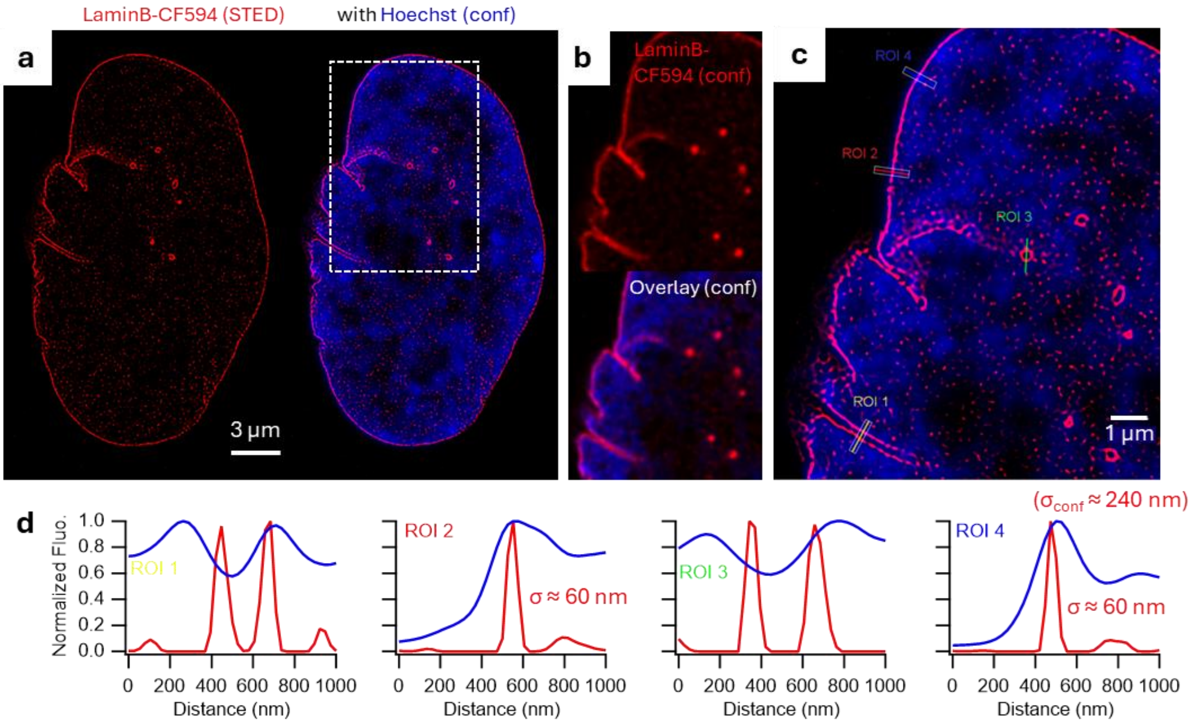

Figure S6: Fixed and permeabilized HeLa cells co-stained with antibodies against LaminB coupled with CF594 and Hoechst. (**a**) LaminB-CF594 STED image of a cell (red, left) and overlay with confocal image of Hoechst (blue, right). (**b**) Zoom of the same cell with Lamin B-CF594 confocal image (red, top) and the overlay with Hoechst (bottom). (**c**) Same zoom in with the STED image LaminB-CF594 and multiple ROIs. (**d**) Line profile of each ROIs. σ is the FWHM of each ROIs and corresponds to the thickness of the Lamins B layer.

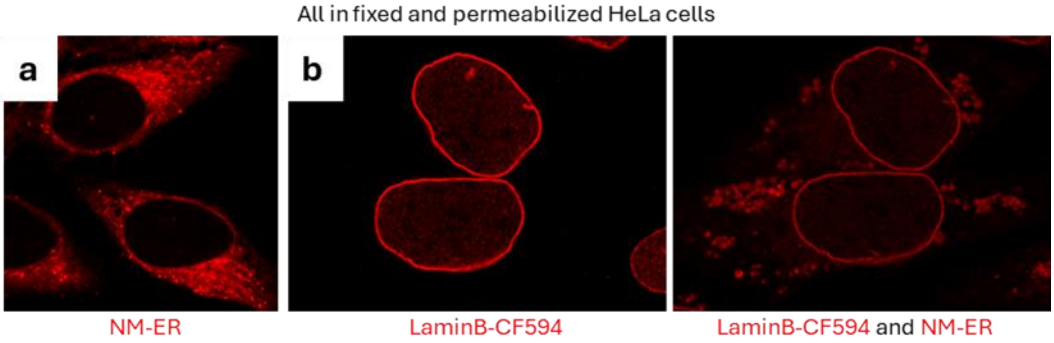

Figure S7: Fixed and permeabilized HeLa cells co stained with NM-ER and LaminB-CF594. HeLa cells were fixed, permeabilized and stained with (**a**) up to 10 µM of NM-ER to reach a good signal of fluorescence or (**b**) antibodies against LaminB conjugated to CF594 (left image). The same amount of NM-ER fluorophore as in (**a**) was added to (**b**) with a loss of signal from CF594 and a different staining pattern in the ER of NM-ER.

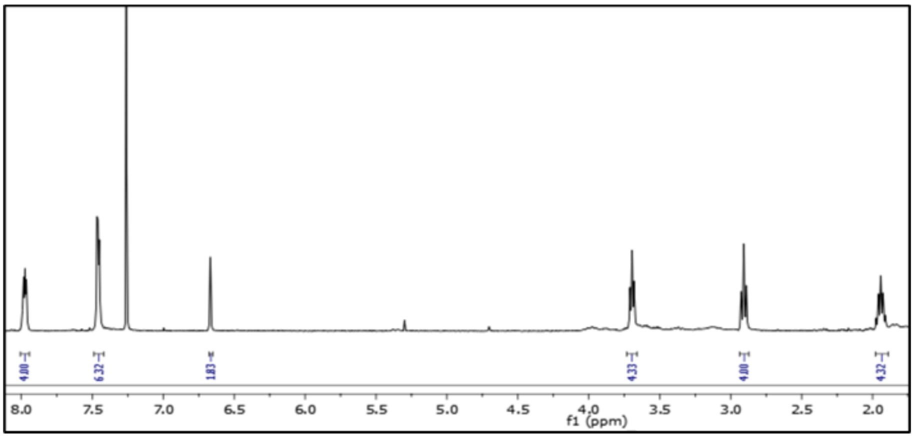

Figure S8. 400 MHz ^{1}H NMR spectrum of **1** in $CDCl_3$.
